# Subsequent ileal stent placement for synchronous small-bowel obstruction through endoscopic ultrasound-guided ileocolostomy with a lumen-apposing metal stent

**DOI:** 10.1055/a-2608-0673

**Published:** 2025-06-13

**Authors:** Kyong Joo Lee, Se Woo Park, Dong Hee Koh

**Affiliations:** 1 366256 Division of Gastroenterology, Department of Internal Medicine, Hallym University Dongtan Sacred Heart Hospital, Hwaseong, South Korea

Peritoneal carcinomatosis is a severe complication of advanced gastrointestinal cancer, often leading to malignant bowel obstructions (MBOs) at multiple synchronous or metachronous points, with distressing symptoms such as frequent vomiting 1 2 . Surgical management of MBO presents significant challenges in these patients 3 . Here, we report a case of stepwise endoscopic management of MBO using endoscopic ultrasound (EUS)-guided ileocolostomy with a lumen-apposing metal stent (LAMS), followed by metal stent placement through the LAMS for synchronous small-bowel obstruction.

A 64-year-old woman with locally advanced pancreatic cancer with multiple metastases presented with persistent vomiting owing to peritoneal carcinomatosis. Abdominal computed tomography (CT) scanning revealed marked small-bowel dilatation ( Fig. 1 **a** ), with distal ileal obstruction ( Fig. 1 **b** ). Given her unsuitability for surgery, an initial attempt at enteral stent placement via colonoscopy was made but was unsuccessful owing to limited advancement of the scope. Subsequently, EUS-guided ileocolostomy ( Video 1 ) was performed using an electrocautery-enhanced LAMS (Niti-S HOT SPAXUS; Taewoong Medical, Gyeonggi-do, Korea) and the free-hand technique. Upon successful deployment, a substantial volume of liquid fecal material drained into the sigmoid colon through the LAMS.

**Fig. 1:**
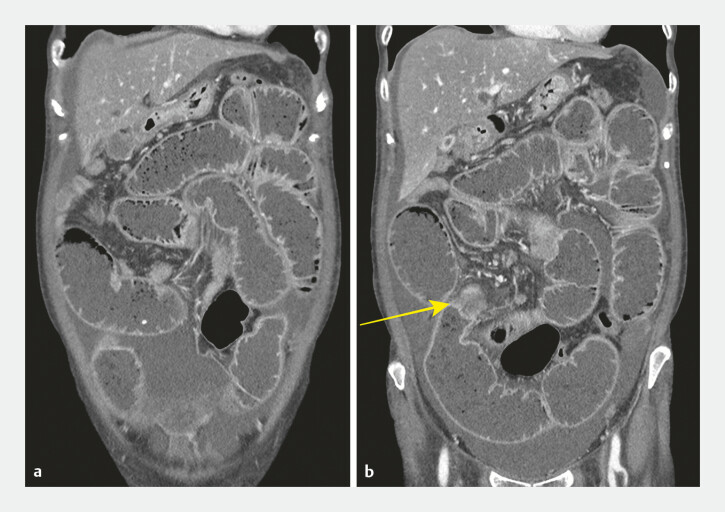
Initial computed tomography images showing: **a** marked dilatation of the entire small intestine, accompanied by mild ascites; **b** a distinct stricture (yellow arrow) in the distal ileum, likely due to recurrent peritoneal carcinomatosis.

Stepwise endoscopic ultrasound-guided ileocolostomy is performed using a lumen-apposing metal stent (LAMS), followed by small-bowel stent placement through the LAMS.Video 1

The patient was readmitted 1 month later with abdominal distension and frequent vomiting. A follow-up CT showed progression of the peritoneal carcinomatosis, with synchronous small-bowel obstruction in the mid ileum ( Fig. 2 **a** ). Colonoscopy was performed ( Video 1 ), and access through the LAMS allowed identification of a distal ileal obstruction. After the obstruction had been cannulated and contrast injected to delineate the occluded segment, a guidewire was placed. Subsequently, a 6-cm uncovered self-expandable metal stent (Niti-S duodenal stent; Taewoong Medical) was successfully deployed ( Fig. 2 **b** ), leading to clinical improvement, and the patient was discharged from hospital.

**Fig. 2:**
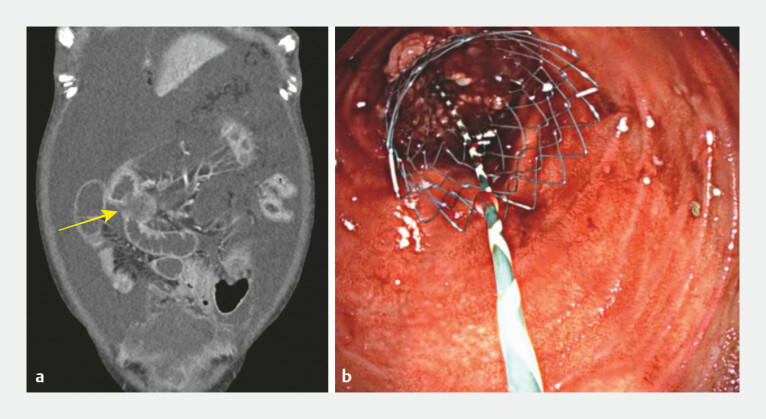
Images of colonoscopic small-bowel stenting for synchronous ileal obstructions showing: **a** on follow-up computed tomography scan, progression of peritoneal carcinomatosis with synchronous small-bowel obstruction (yellow arrow) in the mid ileum; **b** on colonoscopy, a successfully deployed 6-cm uncovered self-expandable metal stent.

This case highlights a novel, minimally invasive, stepwise endoscopic approach for multifocal MBO, demonstrating the feasibility of EUS-guided ileocolostomy with a LAMS to facilitate subsequent interventions, including stent placement, for synchronous or metachronous small-bowel obstructions.

Endoscopy_UCTN_Code_TTT_1AS_2AZ

## Notes

Endoscopy E-Videos https://eref.thieme.de/e-videos
*E-Videos* is an open access online section of the journal *Endoscopy* , reporting on interesting cases and new techniques in gastroenterological endoscopy. All papers include a high-quality video and are published with a Creative Commons CC-BY license. Endoscopy E-Videos qualify for HINARI discounts and waivers and eligibility is automatically checked during the submission process. We grant 100% waivers to articles whose corresponding authors are based in Group A countries and 50% waivers to those who are based in Group B countries as classified by Research4Life (see: https://www.research4life.org/access/eligibility/ ). This section has its own submission website at https://mc.manuscriptcentral.com/e-videos .
